# A New Technique for Broadband Matching of Open-Ended Rectangular Waveguide Radiator

**DOI:** 10.3390/s23229176

**Published:** 2023-11-14

**Authors:** Ji-Won Heo, Songyuan Xu, Erdenesukh Altanzaya, Qiongyue Zhang, Chan-Soo Lee, Bierng-Chearl Ahn, Jae-Hyeong Ahn, Seong-Gon Choi

**Affiliations:** School of Electric and Computer Engineering, Chungbuk National University, Cheongju 28644, Republic of Korea; gjwldnjs131@naver.com (J.-W.H.); xusongyuan1222@gmail.com (S.X.); eeric9655@outlook.com (E.A.); qy1213z@126.com (Q.Z.); direct@chungbuk.ac.kr (C.-S.L.); bician@chungbuk.ac.kr (B.-C.A.)

**Keywords:** waveguide open-end radiator, rectangular waveguide, impedance matching, capacitive matching

## Abstract

The maximum reflection at an open end of a standard rectangular waveguide is about −10 dB in its operating frequency range. It is often used without matching. For critical applications, it is desirable to further reduce the reflection coefficient. In this paper, a new technique is presented for the broadband impedance matching of an open-ended rectangular waveguide. The proposed technique employs three thin capacitive matching elements placed at proper intervals via a low-loss dielectric material. The capacitance of, and distance between, the matching elements are optimized for broadband impedance matching using a simulation tool. Based on the proposed technique, two design examples are presented for the matching of a WR75 waveguide radiator. A reflection coefficient of less than −16 dB and −20 dB has been achieved over a ratio bandwidth of 2.13:1 and 1.62:1, respectively.

## 1. Introduction

Since the first experiment in 1900 by Flemming [1] and the first scientific theory in 1938 [2], the rectangular waveguide open-end radiator, as one of the canonical radiating elements, has been used in a wide range of applications, including near-field antenna measurement [3,4,5,6,7,8,9], dielectric material characterization [10,11], electric field measurement [12,13], array antennas [14,15,16], electric field probe calibration [17], feeding a reflector antenna [18], non-destructive testing [19,20,21], imaging sensors [22] and the excitation of various radiating elements [23,24,25,26]. A comprehensive review of various open-ended waveguides has been presented in [27].

The aperture reflection of a rectangular waveguide open end strongly depends on the ratio of the narrow-wall height (*b*) to the broad-wall width (*a*) and weakly depends on the waveguide wall thickness (*t*). The smaller the *b*/*a* ratio, the larger the aperture reflection. In a standard rectangular waveguide ranging from the largest WR2300 (584.2 × 282.1 mm^2^; 0.32−0.49 GHz) to the smallest WR1(0.254 × 0.127 mm^2^; 750−1100 GHz), *b*/*a* and *t*/*a* range from 0.406 to 0.512 and from 0.0081 to 0.467, respectively. The maximum reflection is 0.313 (−10.1 dB) for *b*/*a* = 0.406 and 0.225 (−13.0 dB) for *b*/*a* = 0.512 [9].

The level of reflection in an unmatched open end of a rectangular waveguide might be acceptable in some applications. For other applications, it is desirable for an open-ended waveguide radiator to have a smaller reflection coefficient over a broad frequency range. With reduced aperture reflection, accuracy is improved in measurement applications and efficiency is increased in array antenna applications.

Broadband impedance matching of the open-ended rectangular waveguide (OEG) is of the highest importance in precision measurement applications, where it is a usual practice to use metrology-grade accessories and instruments. The OEG’s improved impedance matching would be beneficial in antenna near-field measurements, the precise generation and measurement of electromagnetic fields and material constants measurements using the free-space method. Improved matching in measurement applications reduces errors arising from imperfections in calibration.

In a large phased array employing open-ended waveguide radiating elements, impedance matching reduces the reflected power, which leads to a significant improvement in power efficiency and a resultant saving on the device’s cooling costs.

**Figure 1:**
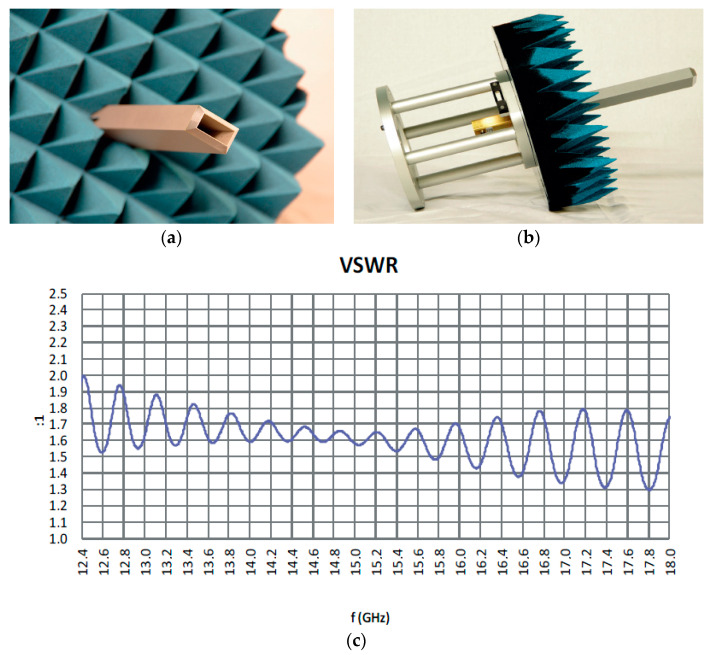
Commercial near-field antenna measurement probe by TTI Norte S.L. Co. [28]: (**a**) front view, (**b**) side view and (**c**) probe’s input VSWR at the coaxial input port.

In the following, we will describe in some detail a need for better impedance matching of an OEG probe in the antenna near-field measurements. A commercial near-field waveguide probe by TTI Norte S.L. Co. uses a WR62 waveguide (15.80 × 7.90 mm^2^) open end [28]. The aperture walls are chamfered as shown in Figure 1a. An absorber collar is placed around the probe to reduce wave reflection and scattering from the probe fixture, as shown Figure 1b. The probe’s input VSWR at the coaxial port is shown in Figure 1c and ranges from 1.3 to 2.0, or the reflection coefficient from −17.7 dB to −9.5 dB with multiple maxima and minima caused by the reflection between the probe aperture and the coaxial-to-waveguide transition.

In near-field antenna measurements, it is important to reduce the wave reflection and scattering by the scanning probe in order to minimize the effects of multiple reflections between the probe and the antenna under test (AUT), which is complicated and difficult to calibrate out [29]. Reflection from the probe aperture caused by impedance mismatch is another contributor to the multiple-reflection effects.

The reflection coefficients of a probe and an AUT in antenna near-field measurements are accounted for in a power transfer equation via 1 − |Γ|^2^, where Γ is the reflection coefficient. The reflection coefficient is entered into a classical formula for the probe’s boresight gain by Yaghijan [3], which reads

(1) $$G_{02}=\frac{\pi k^{2}ab}{8{\left(1-|\Gamma |\right)}^{2}\beta /k}{\left|\left[1+\frac{\beta }{k}+\Gamma \left(1-\frac{\beta }{k}\right)\right]{\left(\frac{2}{\pi }\right)}^{2}+C_{0}\right|}^{2}$$

Note that the complex value of the reflection coefficient Γ is used in (1), not just the magnitude. The near-field probe typically operates in the recommended operating frequency range (1.5:1 bandwidth) of a standard rectangular waveguide. Therefore, it is desirable to reduce the reflection coefficient of a waveguide open end as much as possible over a bandwidth greater than 1.5:1.

In view of the problems discussed in the above, this paper presents a new technique for the broadband impedance matching of an open-ended rectangular waveguide. A thorough literature survey reveals that this is still an open problem. By broadband, we mean the recommended frequency range (1.5:1) of a standard rectangular waveguide and beyond. A comprehensive review of existing works on this issue is presented below.

A simple method for the impedance matching of an open-ended rectangular waveguide is to load the aperture with a dielectric plug. Ivanchenko and co-workers used a Teflon^®^ (dielectric constant *ε_r_* = 2.04) plug in a WR90 waveguide aperture, with fourteen dielectric cylinders of *ε_r_* = 3.8 embedded in the plug [30]. They obtained a reflection coefficient <−15 dB at 9.5−10.5 GHz (1.11:1 bandwidth). Zhang and co-workers used a dielectric slab (*ε_r_* = 2.7, thickness *t* = 0.5 mm) inside a circular waveguide (diameter = 12 mm) and close to the aperture. The aperture was equipped with a slot and a choke that were employed for radiation pattern control. They reduced the reflection coefficient to −20 dB at 17.0−19.5 GHz (1.15:1 bandwidth) [31]. The unmatched reflection coefficient ranges from −11.5 to −7.5 dB in the same frequency band.

In a phased array application, it is necessary to obtain good impedance matching in a waveguide radiator operating over large scan angles. An inductive iris matching technique has been employed for this application [32,33]. Van Schaik used an inductive iris on a 35.0 × 11.4 mm^2^ aperture with a polythene dielectric sheet (*ε_r_* = 2.3, *t* = 5 mm) placed outside the waveguide at 5 mm distance from the aperture to obtain reflection <−10 dB at 5.4−5.9 GHz for scan angles up to 60° [32].

More recent studies on this topic have been concerned with the impedance matching of an evanescent waveguide aperture. Ludlow and Fusco placed a dielectric slab (*ε_r_* = 6.15, *t* = 6.36 mm) at 0.7-mm in front of a 55.0 × 27.5 mm^2^ waveguide (*f_c_*_TE10_ = 2.73 GHz) aperture to obtain −10-dB reflection at 2.13−2.70 GHz [34]. Their radiator is more like a cavity antenna than a waveguide radiator. A coaxial probe is placed at 23 mm from a back short and at 24 mm from the aperture. The probe sets up an aperture field via proximity coupling, and the dielectric slab changes the aperture admittance so that aperture matching occurs below the waveguide cutoff. The method of placing a dielectric slab in front of an aperture has long been employed for wide-scan angle impedance matching in waveguide phased arrays [35].

Ludlow and co-workers presented an impedance matching method for an open-ended evanescent-mode rectangular waveguide, using a conducting post in the aperture which is coupled directly to a feeding coaxial probe [36], or via three conducting posts placed between a feeding probe and a conducting post in the aperture [37]. The aperture in [36] works at 4.43−4.57 GHz in a waveguide having a cutoff at 6.56 GHz. The aperture of [37] is realized in a waveguide with a cutoff at 2.72 GHz and has a reflection coefficient of less than −10 dB at 2.30−2.68 GHz.

Ludlow and co-workers presented the concept of distributed coupled resonators for the impedance matching of an evanescent-mode rectangular waveguide radiator [38]. An aperture containing a dielectric slab is excited by two dielectric plugs placed between a feeding coaxial probe and the dielectric slab in the aperture. The dielectric plugs and the waveguide section between them form two coupled resonators. They achieved a ratio bandwidth of 1.23 (2.00−2.45 GHz) for a reflection coefficient <−10 dB using a waveguide with a cutoff at 2.58 GHz.

Other impedance matching methods for a below-cutoff aperture include the use of a negative permeability material [39], an electromagnetic metamaterial [40,41,42,43], a radiator–filter combined structure [44,45,46,47]. Except for the radiator–filter combined structure, the input reflection coefficient is on the level of −10 dB, not −20 dB. These methods tend to yield a narrow-band matching.

A particular challenge in OEG impedance matching is to achieve a large matching bandwidth. In all of the previous studies, it has been difficult to obtain a good impedance matching over a large bandwidth, for example, over more than an octave bandwidth. In this paper, we present a technique for the broadband impedance matching of an open-ended rectangular waveguide radiator using capacitive elements. This is the first time that the capacitive matching technique has been applied to solve this problem. The novelty of the proposed technique is in using printed capacitive elements spaced by a low dielectric constant material for a simple, low-cost implementation of the broadband impedance matching of the rectangular waveguide open-end radiator. The following are the major contributions of this paper.

- OEG impedance matching over more than an octave bandwidth;
- Achieving −20 dB reflection in the standard waveguide band;
- OEG matching with a series of printed-circuit capacitive elements precisely placed inside a waveguide via a low-dielectric-constant material.

We will show the proposed technique using two design examples—a broadband design and a low-reflection design. The two examples have been obtained using a powerful modern electromagnetic simulation tool (CST Studio Suite^TM^ V2023). The authors believe that the simulation tool used in this paper is accurate enough to fully demonstrate the proposed technique. All the details—the structure, technique, simulation and dimensions—are provided, so that anyone can easily reproduce the result. In the next section, we will present the proposed technique and design examples based on it.

Firstly, the reflection coefficient of an empty OEG is analyzed. Secondly, the geometry of the proposed matching structure is presented, along with its equivalent circuit representation, followed by the analysis of the circuit property of the capacitive matching element. Thirdly, two design examples of the OEG matching using the proposed technique are presented. Finally, this study is compared with previous research, followed by conclusions.

## 2. Aperture Matching of the Rectangular Waveguide Open End

**Figure 2:**
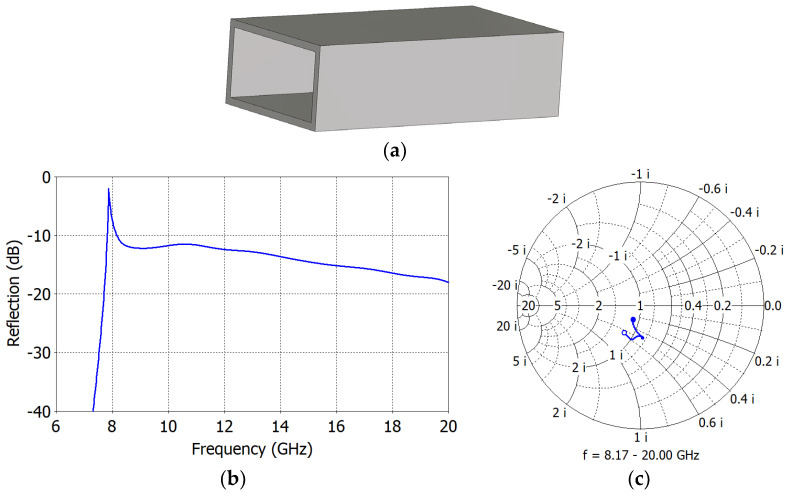
WR75 waveguide open-end radiator (**a**), its reflection coefficient (**b**) and its aperture admittance (**c**).

Figure 2a shows an open-ended WR75 waveguide with broad-wall width *a* = 19.05 mm, narrow-wall width *b* = 9.53 mm and wall thickness *t* = 1.27 mm. The cutoff frequency *f_c_* and cutoff wavelength *λ_c_* of the TE_10_ mode in a rectangular waveguide filled with a material of dielectric constant *ε_r_* are given by

(2) $$f_{c}(\mathrm{GHz})=\frac{300}{2a\sqrt{\varepsilon_{r}}};\lambda_{c}=\frac{2a}{\sqrt{\varepsilon_{r}}}$$

where *a* is the broad-wall width in mm. In a WR75 waveguide with *ε_r_* = 1, the fundamental TE_10_ mode cutoff is at 7.87 GHz. Figure 2b shows the simulated aperture reflection coefficient Γ*_A_* of a WR75 waveguide radiator. It decreases steadily from −10 dB at 8.17 GHz (≡ *f*_1_ = 1.038 *f_c_*) to −17.9 dB at 20 GHz (≡ *f*_2_ = 2.54 *f_c_*). Figure 2c shows the normalized aperture admittance *y_A_* (= *g_A_* + *jb_A_*), whose real part *g_A_* and imaginary part *b_A_* range from 0.839 to 1.17 and from 0.253 to 0.587, respectively, at 8.17−20 GHz. The aperture has a positive susceptance *b_A_* presenting a capacitive load to the waveguide. Chamfering the walls of the open end for scattering reduction changes the aperture admittance only slightly.

**Figure 3:**
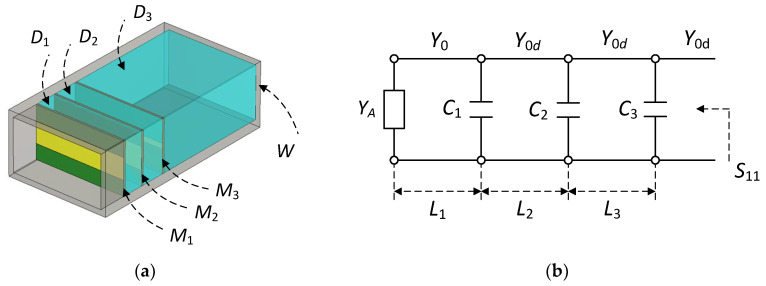
Proposed matching structure (**a**) and its equivalent circuit (**b**). Gray = waveguide, Blue = dielectric matrieral, Green = PCB, Yellow = metal trace.

Figure 3a shows the proposed structure for the broadband matching of an open-ended rectangular waveguide radiator, whose equivalent circuit representation is shown in Figure 3b. The proposed technique utilizes three matching elements *M*_1_, *M*_2_ and *M*_3_ of a shunt capacitive type. *D*_1_ and *D*_2_ are a dielectric material of low dielectric constant (*ε_r_*) employed to support and to precisely position the matching elements *M*_1_, *M*_2_ and *M*_3_. The same dielectric material *D*_3_ fills the waveguide *W*. Without *D*_3_, the level of impedance matching is reduced. Filling the space between the aperture and the first matching element *M*_1_ with a dielectric material also reduces the level of impedance matching. The dielectric constant *ε_r_* is small, so that a coaxial-to-waveguide transition can be designed using the same technique as that for an air-filled waveguide.

In Figure 3b, *Y_A_* is the aperture admittance of the matching elements *M*_1_ to *M*_3_, *Y*_0_ and *Y*_0*d*_ are the waveguide characteristic impedance, *C*_1_ to *C*_3_ are the capacitance, *L*_1_ is the aperture-to-*M*_1_ distance, *L*_2_ and *L*_3_ are the inter-element distance.

The matching elements are implemented in a thin horizontal metal strip symmetrically placed in the waveguide’s *E* plane. They can be constructed in a printed form on a film substrate [48] or on a thin reinforced PTFE laminate [49]. By optimizing the matching element’s capacitance and the distance between the matching elements, one can obtain a broadband impedance matching with a start frequency close to the TE_10_ mode cutoff.

The mechanism behind the all-shunt-capacitive broadband matching of a waveguide aperture is not simple. The shunt capacitance, as well as the aperture admittance, is frequency-dependent. Thus, computer optimization is one of the efficient ways to find a desired solution for broadband matching.

**Figure 4:**
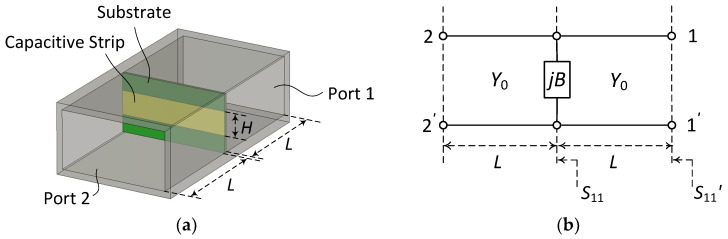
Capacitive matching element in a rectangular waveguide (**a**) and its equivalent circuit representation (**b**).

Firstly, characteristics of the capacitive matching element are studied. Figure 4a shows a capacitive matching element placed inside a rectangular waveguide whose length is 2 *L* plus the matching element’s thickness. Figure 4b shows its equivalent circuit. When the matching element’s thickness is very small relative to the guided wavelength *λ_g_*, it can be approximated as a purely shunt element. An *E*-plane-centered horizontal strip of zero thickness in a rectangular waveguide can be represented by a shunt capacitance as shown in Figure 4b. The normalized susceptance *B*/*Y*_0_ obtained using the variational method is given by [50]

(3) $$\frac{B}{Y_{0}}=4t\left[-\ln(\cos p)+\frac{Q^{2}\sin^{4}p}{1+Q^{2}\cos^{4}p}+{\left(\frac{t}{4}\right)}^{2}{\left(1-3\cos^{2}p\right)}^{2}\sin^{4}p\right]$$

(4) $$p=\frac{\pi H}{2b};t=\frac{b}{\lambda_{g}};\lambda_{g}=\frac{\lambda }{\sqrt{1-{\left[\lambda /(2a)\right]}^{2}}};Q=\frac{1}{\sqrt{1-{\left(b/\lambda_{g}\right)}^{2}}}-1$$

where *B* is the un-normalized shunt susceptance, *Y*_0_ is the characteristic admittance in the equivalent circuit representation of the waveguide, *H* is the strip height, *b* is the waveguide narrow-wall height, *λ_g_* is the guided wavelength, *λ* is the wavelength in vacuum and *a* is the waveguide broad-wall width.

We calculate the scattering parameters *S*_11′_ (=*S*_22′_) and *S*_21′_ (=*S*_12′_) of the structure of Figure 4a and then move the port reference plane to the surface of the matching element to obtain the de-embedded scattering parameters *S_ij_*, i.e.,

(5) $$S_{ij}=S_{ij}{}^{\prime }\exp\left(j\frac{4\pi }{\lambda_{g}}L\right)(i,j=1,2)$$

where *λ_g_* is the guided wavelength given in Equation (4). From the de-embedded scattering parameters, we calculate the normalized susceptance *B*/*Y*_0_ of the capacitive element. The reflection coefficient *S*_11_ at the relocated reference plane of Port 1 shown in Figure 4b is now given by

(6) $$S_{11}=-\frac{jb_{c}}{2+jb_{c}};jb_{c}=\frac{jB}{Y_{0}}$$

since Port 2 is matched when calculating the scattering parameters. In Equation (6), *Y*_0_ is the equivalent characteristic impedance of the TE_10_-mode wave. Note that there is no need to explicitly calculate *Y*_0_, since *b_c_* can be obtained from *S*_11_. For the capacitance calculation, however, the value of *Y*_0_ is required. The un-normalized susceptance *B* is calculated using Equation (6) and the capacitance *C* at frequency *f* is now given by the following equation.

(7) $$C=\frac{B}{2\pi f}$$

**Figure 5:**
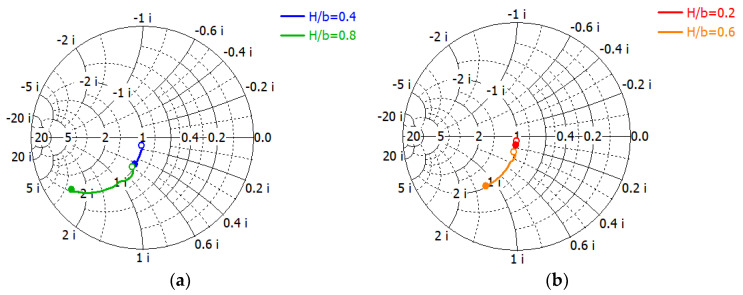
Simulated admittance of a capacitive element in parallel with a matched load in the WR75 waveguide versus frequency. *H* is the capacitive strip height and *b* is the waveguide narrow-wall height: (**a**) for *H*/*b* = 0.4 and 0.8; (**b**) for *H*/*b* = 0.2 and 0.6.

The computed capacitance will be frequency-dependent [50]. In the proposed technique, it is not necessary to calculate the capacitance of the matching elements. Figure 5 shows the loci at 8−16 GHz of the simulated reflection coefficient *S*_11_ (shown in Figure 4b) of a matched load in parallel with a capacitive matching element (with width *a*, height *H*) printed on a film substrate Parylux^®^ TAHS124500 by DuPont^TM^ [48] (substrate: *ε_r_* = 3.4, tan*δ* = 0.0045 at 10 GHz, *h* = 0.0045 mm; strip: copper, *t* = 0.0012) for *H*/*b* ratios 0.2, 0.4, 0.6 and 0.8.

One can identify the normalized susceptance *b_c_* of the capacitive element directly from the admittance Smith chart in Figure 5. Table 1 shows the range of the normalized susceptance *b_c_* versus the *H*/*b* ratio at 8−16 GHz. The range of susceptance values is large enough to cover the aperture susceptance shown in Figure 1c of the WR75 waveguide open-end radiator. We have not used the theoretical Equation (3) to verify the susceptance of the capacitive strip, since our design is not based on the theory but on the computer simulation. A capacitive element in a dielectric-filled waveguide behaves in a similar way and can be analyzed using the same method as described above.

**Figure 6:**
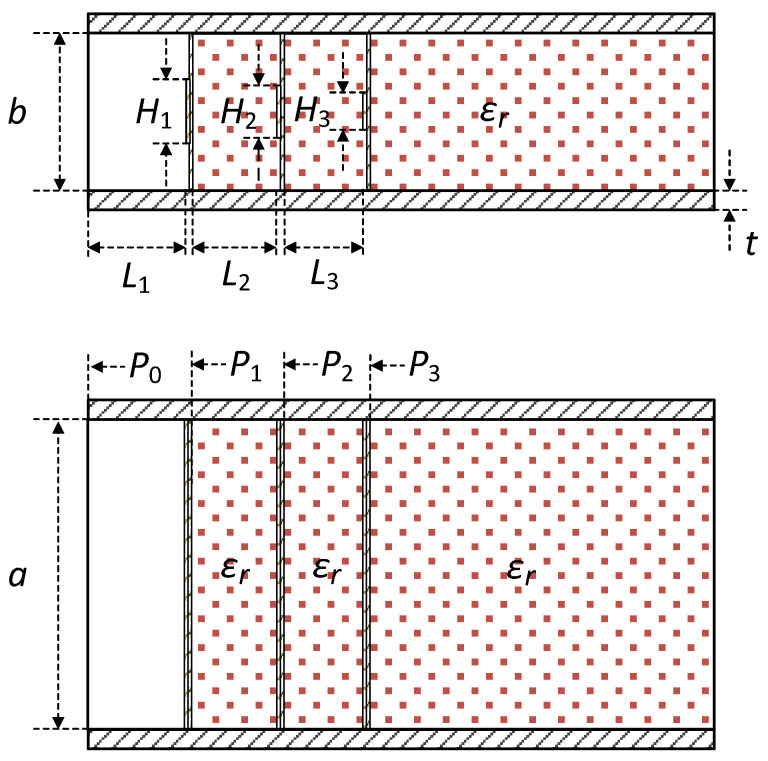
Dimensional parameters of the proposed impedance matching structure.

Figure 6 shows the dimensional parameters of the proposed matching structure, whose meanings are explained in Table 2. The proposed impedance matching structure can be optimized for |*S*_11_| < −20 dB over the recommended operating frequency range of a standard rectangular waveguide (*b* = *a*/2), which we call a ‘Standard-Band Design’. Alternatively, a matching structure can be designed for the widest possible bandwidth over which |*S*_11_| is less than, for example, −16 dB, which we call a ‘Broadband Design’. In Figure 6, *P*_0_ to *P*_3_ refer to the reference planes to be used in the progressive impedance matching analysis.

**Figure 7:**
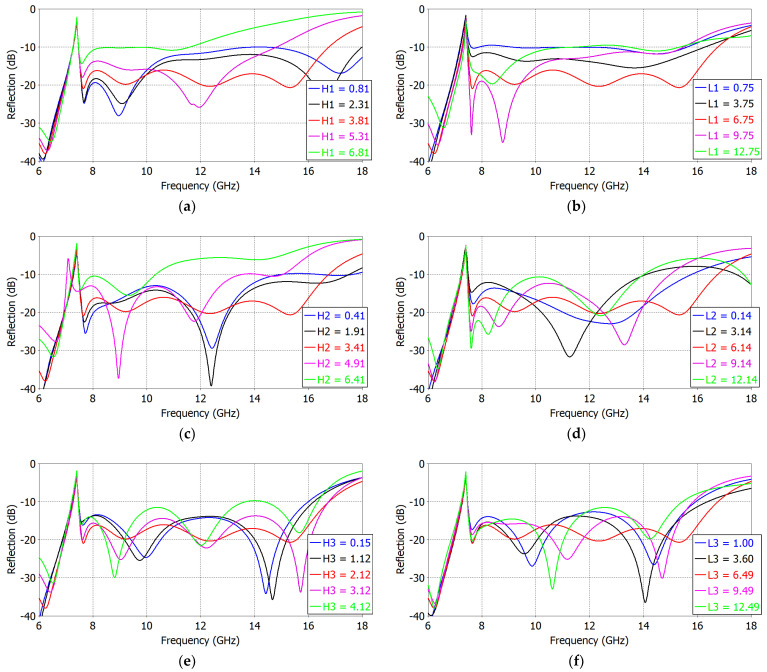
Reflection coefficient versus the capacitive strip heights *H*_1_, *H*_2_, *H*_3_ and the inter-element distances *L*_1_, *L*_2_, *L*_3_ of the first (**a**,**b**), second (**c**,**d**) and third (**e**,**f**) matching elements in the ‘Broadband Design’ of an impedance-matched WR75 waveguide radiator.

Before optimization via a simulation tool, we carried out a series of parametric analyses on the reflection coefficient of the ‘Broadband Design’ of an impedance-matched WR75 waveguide radiator. The results are shown in Figure 7. The dielectric constant *ε_r_* supporting the capacitive elements is 1.13 in the ‘Broadband Design’, so that the TE_10_-mode cutoff is at 7.40. The reflection coefficient rises above −10 dB from 16.74 GHz onwards.

In a WR75 waveguide filled with a material of *ε_r_* = 1.13, the first six TE modes in order of increasing cutoff frequency *f_c_* are TE_10_, TE_20_, TE_01_, TE_11_, TE_21_ and TE_30_, with *f_c_* of 7.40, 14.80, 14.80, 16.55, 20.94 and 22.21 GHz, respectively. Of these modes, the TE_20_, TE_01_ and TE_21_ modes can be suppressed by using structures that are symmetric in the *H*-plane of the waveguide. The TE_11_ and TE_30_ modes, however, can be excited along with the fundamental TE_10_ mode. Therefore, the waveguide’s operating frequency will be greater than the TE_10_-mode cutoff (7.40 GHz) and less than the TE_11_-mode cutoff (16.55 GHz), resulting in a ratio bandwidth of 2.24 (16.55/7.40). In Figure 7, the frequency range of the analysis is set from 6 GHz to 18 GHz.

In Figure 7, we note that the matching element heights *H*_1_, *H*_2_, *H*_3_ and distances *L*_1_, *L*_2_, *L*_3_ between the matching elements have a sensitive effect on the reflection coefficient. The final ‘Broadband Design’ is obtained with an optimum combination of all of these parameters.

Next, an automatic optimization process is employed to find optimum values of *H*_1_, *H*_2_, *H*_3_, *L*_1_, *L*_2_, *L*_3_ and *ε_r_*. The CST Studio Suite^TM^ 2023 offers a set of built-in optimization algorithms, among which, the “Trust Region Framework” has been used in this study. The ranges of frequency and parameter values in the optimization have been obtained from the parametric analysis carried out in the previous step.

**Figure 8:**
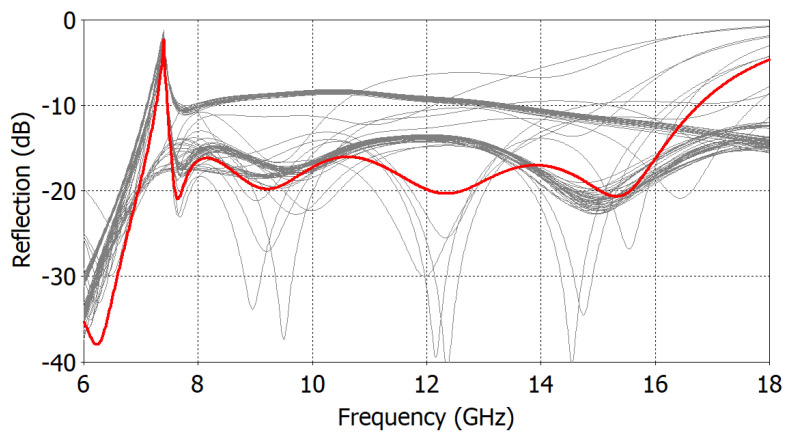
Reflection coefficient variation during an optimization of the ‘Broadband Design’ of an impedance-matched WR75 waveguide radiator. The curve in red is the final converged result.

Figure 8 shows the reflection coefficient variation during an optimization of the ‘Broadband Design’. The curve in red is the reflection coefficient of the final design of the impedance-matched WR75 waveguide radiator, while the curves in gray are intermediate reflection coefficients. As will be shown later in the ‘Standard-Band Design’, one can find an optimum design for a specific frequency range over which the reflection coefficient is less than a specified value. With a given number of the matching elements, the bandwidth will be smaller for a smaller reflection coefficient.

Following the aforementioned procedures, we obtained a second design example—the ‘Broadband Design’ whose dimensions are listed in Table 3. In Table 3, *h* is the substrate thickness and *t* is the metal-trace thickness of the capacitive matching elements.

**Figure 9:**
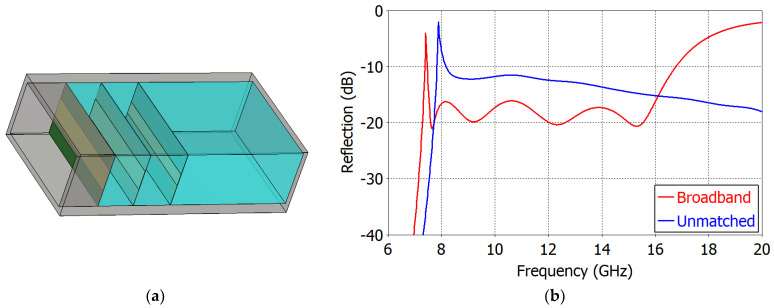
‘Broadband Design’ of an impedance-matched WR75 waveguide radiator: (**a**) structure; (**b**) reflection coefficients of an unmatched WR75 waveguide radiator (in blue) and the ‘Broadband Design’ (in red).

Figure 9a shows the structure of the ‘Broadband Design’, and Figure 9b compares the reflection coefficients of an unmatched radiator and the ‘Broadband Design’. In the unmatched case, the reflection coefficient ranges from −10.0 dB at 8.17 GHz to −18.0 dB at 20 GHz. In the ‘Broadband Design’, the reflection coefficient is less than −16.0 dB at 7.53−16.01 GHz (ratio bandwidth 2.13:1). Filling the waveguide with a material of *ε_r_* = 1.13 lowers the cutoff frequency from 7.87 GHz of the air-filled WR75 waveguide to 7.40 GHz.

**Figure 10:**
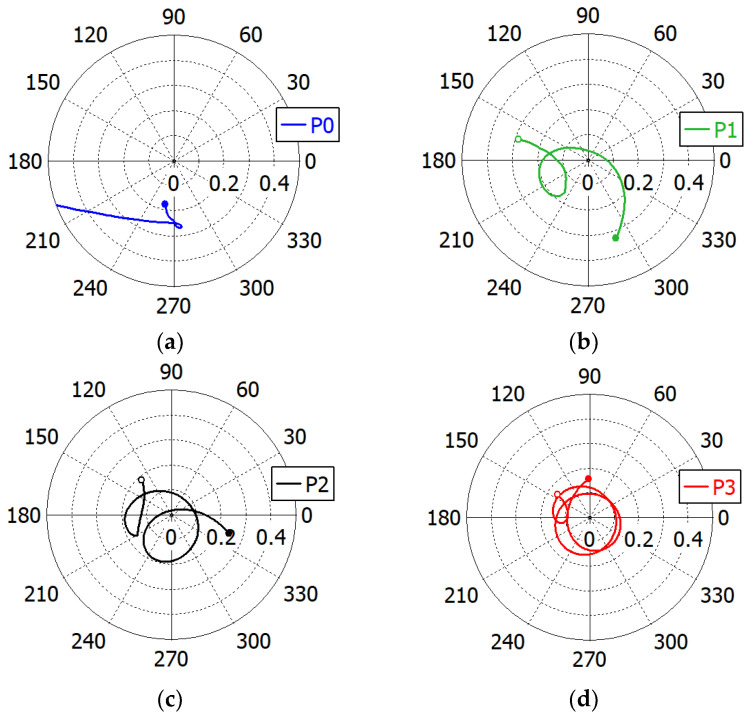
Reflection coefficient loci in the ‘Broadband Design’ of an impedance-matched WR75 waveguide radiator: in the aperture (**a**), just after the first (**b**), the second (**c**) and the third (**d**) matching elements. The start frequency (7.53 GHz) is marked with an open circle and the end frequency (16.01 GHz) with a filled circle.

**Figure 11:**
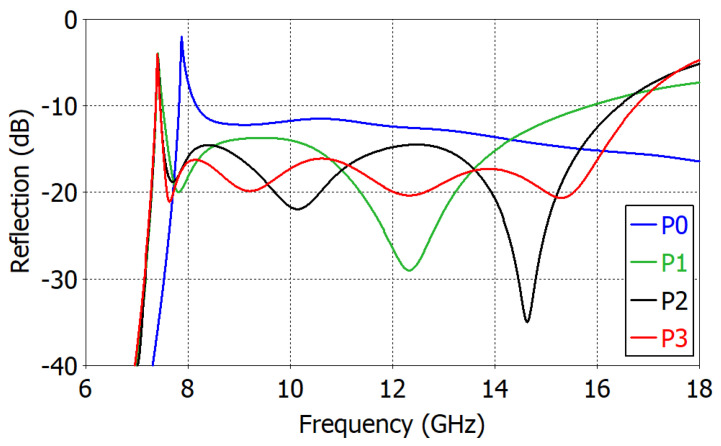
Reflection coefficient magnitude in the ‘Broadband Design’ of an impedance-matched WR75 waveguide radiator: in the aperture (P0), just after the first (P1), the second (P2) and the third (P3) matching elements.

To see the mechanism of the capacitive matching, the complex reflection coefficient of the ‘Broadband Design’ is calculated at 7.53−16.01 GHz in the aperture (on the plane *P*_0_ in Figure 6) and just after each matching element *M*_1_, *M*_2_ and *M*_3_ (planes *P*_1_, *P*_2_ and *P*_3_ in Figure 6, respectively) and is shown in Figure 10. We observe that the range of variation in the reflection coefficient magnitude is progressively reduced after each matching element. Figure 11 shows the change in the reflection coefficient magnitude in dB with the addition of each matching element. The reduction in the reflection coefficient magnitude is not monotonic.

**Figure 12:**
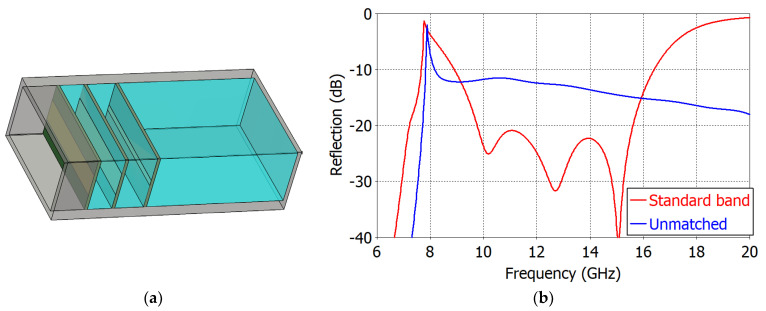
‘Standard-Band Design’ of an impedance-matched WR75 waveguide radiator: (**a**) structure; (**b**) reflection coefficients of an unmatched radiator (in blue) and the ‘Standard-Band Design’ (in red).

Following the same procedures, we obtained a ‘Standard-Band Design’ shown in Figure 12a. The reflection coefficient is shown in Figure 12b. The frequency range of optimization is set from 10 GHz to 15 GHz, which is the recommended operating frequency range of the WR75 waveguide. The target reflection coefficient is set at −20 dB. The smaller reflection coefficient can be set as a target if the required bandwidth is smaller.

**Figure 13:**
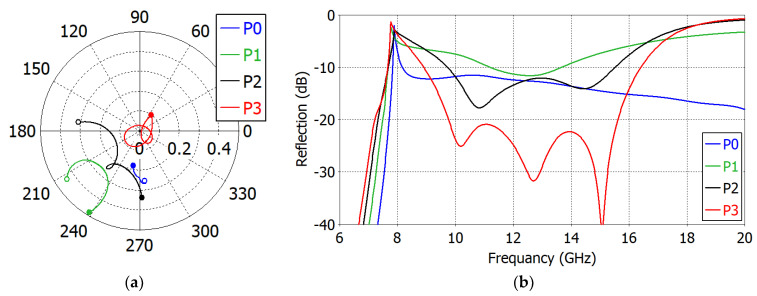
Impedance loci (**a**) and reflection coefficient (**b**) in the ‘Standard-Band Design’ of an impedance-matched WR75 waveguide radiator: in the aperture (P0), just after the first (P1), the second (P2) and the third (P3) matching elements. In (**a**), the start frequency (7.53 GHz) is marked with an open circle and the end frequency (16.01 GHz) with a filled circle.

Table 3 lists the dimensions of the ‘Standard-Band Design’ also. The matching elements are implemented on I-Tera MT40^®^ substrate by Isola Group (substrate: *ε_r_* = 3.45, tan*δ* = 0.0031 @ 10 GHz, *h* = 0.51 mm; strip: copper, *t* = 0.0018) [49]. In the ‘Standard-Band Design’, the reflection coefficient is less than −20 dB at 9.89–15.99 GHz (1.62:1 bandwidth). Figure 13a shows the complex reflection coefficient loci and Figure 13b the reflection coefficient magnitude at planes *P*_0_ to *P*_3,_ with the frequency from 9.89 GHz to 15.99 GHz. In Figure 13b, we observe that the magnitude of the reflection coefficient even increases before the addition of the final matching element *M*_3_. After the final matching element, the reflection coefficient is significantly reduced.

In Table 4, our design is compared with previous studies. As can be seen in Table 4, the new impedance matching technique proposed in this paper delivers a 1.62:1 bandwidth (9.89−15.99 GHz), with reflection <−20 dB comfortably covering the recommended operating frequency range (10−15 GHz) of the WR75 rectangular waveguide. This enables a realization of a near-field measurement probe with improved characteristics. With reflection <−16 dB, the achieved bandwidth is 2.13:1 (7.53−16.01 GHz), offering more than an octave bandwidth which can be useful for wideband/multi-band RF applications and materials measurements.

## 3. Conclusions

This paper proposes, for the first time in a published work, a capacitive impedance matching technique for an open-ended rectangular waveguide radiator. The proposed technique uses a three-section capacitive matching circuit of a shunt type. For easy implementation, the capacitive elements are printed on a film or on a laminated substrate, which are supported and precisely positioned by a low-loss dielectric material. The capacitance of the matching elements is frequency-dependent, and computer-based optimization has been employed to achieve a broadband matching. For improved matching performance, it is necessary to fill the space between the aperture and the first matching element with air, and to fill the space between the matching elements and the waveguide interior with a low-dielectric-constant material. Two design examples based on the proposed method show that an open-ended rectangular waveguide radiator can be matched with a 2.13:1 bandwidth for a reflection coefficient <−16 dB and with a 1.62:1 bandwidth for a reflection coefficient <−20 dB, which is more than enough to cover the recommended operating frequency range of a standard rectangular waveguide. Areas of further improvements in the proposed technique are (1) achieving a reflection coefficient <−20 dB with a ratio bandwidth greater than 2:1 and (2) reducing the reflection coefficient to less than −30 dB in the operating frequency range of a standard rectangular waveguide. Nonetheless, considering the wide-ranging applications of the open-ended rectangular waveguide radiator, we expect that the proposed technique will significantly contribute to the art of the related engineering disciplines.

## Figures and Tables

**Table 1:** Range of the normalized admittance *B*/*Y*_0_ of the capacitive element at 8−16 GHz.

*H*/*b*	Min (*B*/*Y*_0_)	Max (*B*/*Y*_0_)
0.2	0.082	0.162
0.4	0.151	0.517
0.6	0.282	1.214
0.8	0.593	2.651

**Table 2:** Meaning of the dimensional parameters in the proposed impedance matching structure.

Parameter	Meaning
*a*, *b*, *t*	Waveguide broad-wall width, narrow-wall height and wall thickness
*ε_r_*	Dielectric constant of the material supporting the matching elements and filling the waveguide
*H*_1_, *H*_2_, *H*_3_	Strip width of the capacitive matching elements *M*_1_, *M*_2_ and *M*_3_, respectively
*L*_1_	Distance between the matching element *M*_1_ and the waveguide aperture
*L*_2_, *L*_3_	Distances between the matching elements *M*_1_ and *M*_2_, *M*_2_ and *M*_3_, respectively
*P*_0_	Plane of the aperture
*P*_1_, *P*_2_, *P*_3_	Plane right after the matching elements *M*_1_, *M*_2_ and *M*_3_, respectively

**Table 3:** Dimensions (mm) of the proposed impedance matching structure.

Design	*ε_r_*	*H*_1_	*H*_2_	*H*_3_	*L*_1_	*L*_2_	*L*_3_	Substrate
Broadband	1.13	3.81	3.41	2.12	6.75	6.14	6.49	*ε_r_* = 3.4, tan*δ* = 0.0045, *h* = 0.045, *t* = 0.012
Standard Band	1.03	3.92	4.12	1.01	5.75	4.14	4.90	*ε_r_* = 3.45, tan*δ* = 0.0031, *h* = 0.51, *t* = 0.018

**Table 4:** Comparison with previous studies.

Work	Type	Frequency Range *f*_1_−*f*_2_ (GHz)	Reflection (dB)	Ratio Bandwidth *f*_2_/*f*_1_	Complexity
[30]	Dielectric plug	9.5–10.5	−15	1.11	Low
[32]	Inductive iris	5.4–5.9	−10	1.09	Low
[34]	Dielectric slab	2.13–2.70	−10	1.27	Low
[37]	Multiple conducting posts	2.30–2.68	−10	1.17	Medium
[38]	Distributed coupled resonators	2.00−2.45	−10	1.23	Medium
This Work	Printed capacitive elements	9.89−15.99	−20	1.62	Medium
		7.53–16.01	−16	2.13	

## Data Availability

Data are contained within the article.

## References

[1] Ramsay J.-F. (1958). Microwave antenna and waveguide techniques before 1900. Proc. IRE.

[2] Barrow W.-L., Greene F.-M. (1938). Rectangular hollow-pipe radiators. Proc. IRE.

[3] Yaghjian A. (1984). Approximate formulas for the far field and gain of open-ended rectangular waveguide. IEEE Trans. Antennas Propag..

[4] Paulus A., Eibert T.-F. (2023). Fully probe-corrected near-field far-field transformations with unknown probe antennas. IEEE Trans. Antennas Propag..

[5] Sun B., Wen Y., Wang Z. Calibration on open-ended rectangular waveguide from 1GHz to 26.5GHz. Proceedings of the 5th IEEE International Symposium on Microwave, Antenna, Propagation and EMC Technologies for Wireless Communications (MAPE).

[6] Wu D.-I., Kanda M. (1989). Comparison of theoretical and experimental data for the near field of an open-ended rectangular waveguide. IEEE Trans. Electromag. Compat..

[7] Selvan K.-T., Poddar D.-R., Kini K.-R. (2001). Off-axis gain and field in the finite range of an open-ended rectangular waveguide. IEEE Trans. Electromag. Compat..

[8] Kawalko S.-F., Kanda M. (1997). Near-zone gain of open-ended rectangular waveguides. IEEE Trans. Electromag. Compat..

[9] Kim J.-H., Enkhbayar B., Bang J.-H., Ahn B.-C., Cha E.-J. (2010). New formulas for the reflection coefficient of an open-ended rectangular waveguide radiating into air including the effect of wall thickness or flange. Prog. Electromag. Res. M.

[10] Vaccaro M., Al Qaseer M.T., Zoughi R. Dielectric characterization of curved structures using flangeless open-ended waveguide measurement. Proceedings of the IEEE International Instrumentation and Measurement Technology Conference (I2MTC).

[11] Chang C.-W., Chen K.-M., Qian J. (1997). Nondestructive determination of electromagnetic parameters of dielectric materials at X-band frequencies using a waveguide probe system. IEEE Trans. Instrum. Meas..

[12] Ali M., Sanyal S. (2008). FDTD analysis of rectangular waveguide in receiving mode as EMI sensors. Prog. Electromag. Res. B.

[13] Li Q., Gandhi O.-P., Kang G. (2004). An open-ended waveguide system for SAR system validation or probe calibration for frequencies above 3 GHz. Phys. Med. Biol..

[14] Zarifi D., Farahbakhsh A., Zaman A.-U., Kildal P.-S. A high gain ridge gap waveguide fed slot antenna array for 60 GHz applications. Proceedings of the 10th European Conference on Antennas and Propagation (EuCAP).

[15] Fang Y., Lu Z., Yan X. Design of an open-ended waveguide array with a plane radome. Proceedings of the International Conference on Microwave and Millimeter Wave Technology (ICMMT).

[16] Visser H.-J. Planar, faceted and curved array antenna research at TNO physics and electronics laboratory. Proceedings of the Conference on Perspectives on Radio Astronomy: Science with Large Antenna Array.

[17] Ishigami S., Wu I., Sato K.-I., Gotoh K., Matsumoto Y. Calibration of electric-field probe using open-ended waveguides. Proceedings of the IEEE International Symposium on Electromagnetic Compatibility.

[18] Monebi A.M., Otgonbat D., Ahn B.-C., Lee C.-S., Ahn J.-H. (2023). Conceptual design of a semi-dual polarized monopulse antenna by computer simulation. Appl. Sci..

[19] Ghasr M.-T., Kharkovsky S., Zoughi R., Austin R. (2005). Comparison of near-field millimeter-wave probes for detecting corrosion precursor pitting under paint. IEEE Trans. Instrum. Meas..

[20] Abou-Khousa M.-A., Rahman M.-S.-U., Donnell K.-M., Qaseer M.-T.-A. (2023). Detection of surface cracks in metals using microwave and millimeter-wave nondestructive testing techniques—A review. IEEE Trans. Instrum. Meas..

[21] Qaddoumi N.-N., Saleh W.-M., Abou-Khousa M. (2007). Innovative near-field microwave nondestructive testing of corroded metallic structures utilizing open-ended rectangular waveguide probes. IEEE Trans. Instrum. Meas..

[22] Baskakova A., Hoffmann K. (2022). W-Band imaging sensor using a rectangular waveguide structure with choke. IEEE Microw. Wirel. Compon. Lett..

[23] Leung K.-W., So K.-K. (2003). Rectangular waveguide excitation of dielectric resonator antenna. IEEE Trans. Antennas Propag..

[24] Kumar P., Masa-Campos J.-L. (2015). Waveguide fed circular microstrip patch antenna for Ku band applications. Microw. Opt. Technol. Lett..

[25] Yoo I., Smith D.R. (2022). Design of conformal array of rectangular waveguide-fed metasurfaces. IEEE Trans. Antennas Propag..

[26] Trinh-Van S., Thi T.N., Yang Y., Lee K.-Y., Jung K.-Y., Hwang K.C. (2019). High-gain waveguide-fed circularly polarized spidron fractal aperture antenna. Appl. Sci..

[27] Gardiol F.E., Hawks P.W. (1985). Open-ended waveguides: Principles and applications. Advances in Electronics and Electron Physics.

[28] TTI Norte Open-Ended Rectangular Waveguide Probe (OE-RWP). https://www.ttinorte.es/wp-content/uploads/2019/08/OE-RWP_Open-Ended-Rectangular-Waveguide.pdf.

[29] IEEE Antennas and Propagation Society (2012). IEEE Recommended Practice for Near-Field Antenna Measurements.

[30] Ivanchenko I., Khruslov M., Plakhtiy V., Popenko N., Ronnow D. (2016). X-band aperture antenna with hybrid dielectric inserts. Prog. Electromag. Res. C.

[31] Zhang L.-J., Choi S.-G., Ahn B.-C., Bang J.-H., Kim D.H., Choi Y.-T. (2016). Simplified feed prime focus reflector antenna. Microw. J..

[32] Schaik H. (1978). The performance of an iris-loaded planar phased-array antenna of rectangular waveguides with an external dielectric sheet. IEEE Trans. Antennas Propag..

[33] Ghen C. Octave band waveguide radiators for wide-angle scan phased arrays. Proceedings of the Antennas and Propagation Society International Symposium.

[34] Ludlow P., Fusco V. (2012). Increased bandwidth evanescent open-ended waveguide antenna design using the imaginary smith chart. IEEE Trans. Antennas Propag..

[35] Magill E., Wheeler H. (1966). Wide-angle impedance matching of a planar array antenna by a dielectric sheet. IEEE Trans. Antennas Propag..

[36] Ludlow P., Fusco V. Matching evanescent open-ended waveguide antennas using the imaginary smith chart. Proceedings of the 5th European Conference on Antennas and Propagation (EUCAP).

[37] Ludlow P., Fusco V., Goussetis G., Zelenchuk D.-E. (2013). Applying band-pass filter techniques to the design of small-aperture evanescent-mode waveguide antennas. IEEE Trans. Antennas Propag..

[38] Ludlow P., Fusco V., Goussetis G., Zelenchuk D.E. (2013). Small aperture evanescent-mode waveguide antenna matched using distributed coupled resonators. Electron. Lett..

[39] Hrabar S., Bartolic J., Sipus Z. (2005). Waveguide miniaturization using uniaxial negative permeability metamaterial. IEEE Trans. Antennas Propag..

[40] Samad M.-A., Hamid A.-K. Miniaturization of waveguide antenna using square/circular arrays of SRR. Proceedings of the 5th International Conference on Electronic Devices, Systems and Applications (ICEDSA).

[41] Ouda M., Abutahoun N. (2013). Rectangular waveguide radiator miniaturization using electromagnetic infinity-shaped metamaterial resonator. IUG J. Nat. Eng. Stud..

[42] Yu B., Yang J., Song Y., Wang Z., Zhang T., Yan B., Xu R. (2023). Terahertz metamaterial waveguide with I-shaped resonators for phase and absorption modulation. Photonics.

[43] Jalali M., Sedghi T., Shokri M. A novel metamatarial SRR for waveguide antenna. Proceedings of the Mediterrannean Microwave Symposium (MMS).

[44] Singhal D., Dhwaj K. (2022). Dielectric resonator-based evanescent-mode waveguide filtering antenna. IEEE Antennas Wirel. Propag. Lett..

[45] Sánchez-Escuderos D., Ruiz-Garnica J., Baquero-Escudero M., Soto P., Boria V.-E., Toso G., Angeletti P., Guglielmi M. (2019). Evanescent-mode ridge-waveguide radiating filters for space applications. IEEE Trans. Antennas Propag..

[46] Mahmud R.-H., Salih I.H., Shang X., Skaik T., Wang Y. (2023). A filtering waveguide aperture antenna based on all-resonator structures. Microw. Opt. Technol. Lett..

[47] Hu Z., Wang S., Tu H., Li Y., Yu Y., Wu D.-L. (2021). Coaxial probe-fed open-ended waveguide antenna based on sand casting process with filtering characteristics and all-in-one structure. Int. J. RF Microw. Comput.-Aided Eng..

[48] DuPont Electronics & Industrial EI 10109 Pyralux TAS. https://www.dupont.com/electronics-industrial/pyralux-ta-tas.html.

[49] Isola I-Tera MT40. https://www.isola-group.com/pcb-laminates-prepreg/i-tera-mt40-rf-mw/.

[50] Marcuvitz N. (1985). Waveguide Handbook.

